# Molecular Ecology of Pyrethroid Knockdown Resistance in *Culex pipiens pallens* Mosquitoes

**DOI:** 10.1371/journal.pone.0011681

**Published:** 2010-07-21

**Authors:** Lin Chen, Daibin Zhong, Donghui Zhang, Linna Shi, Guofa Zhou, Maoqing Gong, Huayun Zhou, Yan Sun, Lei Ma, Ji He, Shanchao Hong, Dan Zhou, Chunrong Xiong, Chen Chen, Ping Zou, Changliang Zhu, Guiyun Yan

**Affiliations:** 1 Department of Pathogen Biology, Nanjing Medical University, Nanjing, Jiangsu, China; 2 Key Laboratory of Modern Pathogen Biology of Jiangsu Province, Nanjing, Jiangsu, China; 3 Program in Public Health, University of California Irvine, Irvine, California, United States of America; 4 Shandong Institute of Parasitic Diseases, Jining, Shandong, China; 5 Jiangsu Institute of Parasitic Diseases, Wuxi, Jiangsu, China; Direccion General de Epidemiologia, Peru

## Abstract

Pyrethroid insecticides have been extensively used in China and worldwide for public health pest control. Accurate resistance monitoring is essential to guide the rational use of insecticides and resistance management. Here we examined the nucleotide diversity of the *para*-sodium channel gene, which confers knockdown resistance (*kdr*) in *Culex pipiens pallens* mosquitoes in China. The sequence analysis of the *para*-sodium channel gene identified L1014F and L1014S mutations. We developed and validated allele-specific PCR and the real-time TaqMan methods for resistance diagnosis. The real-time TaqMan method is more superior to the allele-specific PCR method as evidenced by higher amplification rate and better sensitivity and specificity. Significant positive correlation between *kdr* allele frequency and bioassay-based resistance phenotype demonstrates that the frequency of L1014F and L1014S mutations in the *kdr* gene can be used as a molecular marker for deltamethrin resistance monitoring in natural *Cx. pipiens pallens* populations in the East China region. The laboratory selection experiment found that L1014F mutation frequency, but not L1014S mutation, responded to deltamethrin selection, suggesting that the L1014F mutation is the key mutation conferring resistance to deltamethrin. High L1014F mutation frequency detected in six populations of *Cx. pipens pallens* suggests high prevalence of pyrethroid resistance in Eastern China, calling for further surveys to map the resistance in China and for investigating alternative mosquito control strategies.

## Introduction

Current disease vector control strategy is based primarily on the application of insecticides. Pyrethroid insecticides have become by far the most commonly used class of insecticides for indoor residual spray and bednet impregnation due to the low mammalian toxicity and rapid knockdown effect on insects. However, excessive and continuous application of insecticides has caused the development and spread of insecticide resistance, which has become a major obstacle to the control of mosquito-borne diseases [1], [2].

Pyrethroid insecticides bind to insect *para*-sodium channels and lock them in an open state. Disrupting the transmembrane potential of neurons cause insect paralysis and death. Target site insensitivity due to point mutations in the *para*-sodium channel is an important mechanism of resistance to pyrethroid insecticides in various insect species [3]–[15]. In African malaria vector *Anopheles gambiae*, two non-synonymous point mutations in the *para*-sodium channel gene were found associated with knockdown resistance (*kdr*) to pyrethroid insecticides [11]. The mutation detected in West African *A. gambiae* populations results in substitution of leucine by phenylalanine at position 1014 (L1014F), whereas the mutation in East African populations leads to substitution of leucine by serine (L1014S) [11], [16]–[21]. Further, both resistance alleles have been reported to co-occur in *A. gambiae* populations in Nigeria, Cameroon, Equatorial Guinea, Gabon, Uganda, Kenya and Tanzania, Malawi and Mozambique [22], and in *A. arbiensis* in Burkina Faso and Sudan [12], [23].

*Culex* mosquitoes also exhibit high resistance to pyrethroid insecticides [24], [25]. *Culex* mosquitoes are important vector of lymphatic filariasis and several viral pathogens, including St. Louis encephalitis, West Nile encephalitis, Eastern equine encephalitis, Venezuelan equine encephalitis and Japanese encephalitis [26], [27]. The most prevalent *Culex* species in China is *Cx. pipiens pallens*, and resistance to pyrethroid insecticides was reported [24]. Among *Cx. pipiens pallens* populations in China, target insensitivity is a major cause of resistance to pyrethroids. In particular, Song and colleagues reported the L1014F *kdr* allele in *Cx. pipiens pallens* from northern China [14]. In comparison, two forms of *kdr* mutations (L1014F and L1014S) were found in *Cx. pipiens* complex mosquitoes in the US [28]. However, information on the diversity and role of *kdr* alleles in pyrethroid resistance in *Cx. pipiens pallens* is lacking. In this study, we aimed to determine the genetic diversity of the *kdr* gene in natural populations in Eastern China, and to examine the evolutionary response of *kdr* alleles to insecticide selection to illustrate the role of *kdr* alleles in pyrethroid resistance. Martinez-Torres et al. (1999) and Song et al. (2007) reported allele-specific PCR (AS-PCR) methods for detecting L1014F mutation in *Cx. pipiens* complex and *Cx. pipiens pallens* [8], [14]. In the present study we compared the sensitivity and specificity of AS-PCR method to direct sequencing, and developed a highly sensitive and reliable TaqMan probe method that can detect multiple forms of *kdr* alleles in *Cx. pipiens pallens*.

## Results

### Deltamethrin susceptibility bioassay

The mortality rates of *Cx. pipiens pallens* mosquitoes after one hour exposure to diagnostic 0.05% deltamethrin and followed by 24-hour recovery period are given in Table 1. Mortality rates varied significantly among the six populations (ANOVA; *F_5,18_* = 199.98, *P*<0.0001), ranging from 20.2% (Tangkou population) to 78.6% (Nanjing). According to the WHO criteria that an insect population is classified as resistant when mortality rate is below 80%, the six *Cx. pipiens pallens* populations tested here can be classified as resistant to deltamethrin.

**Table 1:** *Culex pipiens pallens* mosquito mortality rate in standard WHO deltamethrin resistance bioassay.

Population	Number of replicates	Total number of mosquitoes used	Mortality percentage (95% confidence interval)
Tangkou	4	99	20.2 (17.4–22.9)
Qingdao	4	104	33.7 (30.9–36.5)
Huaibei	4	96	43.4 (39.8–46.9)
Weishan	4	93	58.1 (54.4–61.8)
Wuxi	4	97	65.8 (63.1–68.9)
Nanjing	4	103	78.6 (76.6–80.6)

### Optimization of *kdr* mutation diagnostic assays

A 521-bp fragment of the sodium channel gene was sequenced from 97 *Cx. pipiens pallens* individuals of Wuxi population. The wildtype codon sequence is TTA in the 1014 site. We detected two non-synonymous mutations in this site: TTA - TTT and TTA - TCA. The first mutation resulted in replacement of leucine by phenylalanine (L1014F) whereas the second mutation leads to substitution of leucine by serine (L1014S).

**Figure 1:**
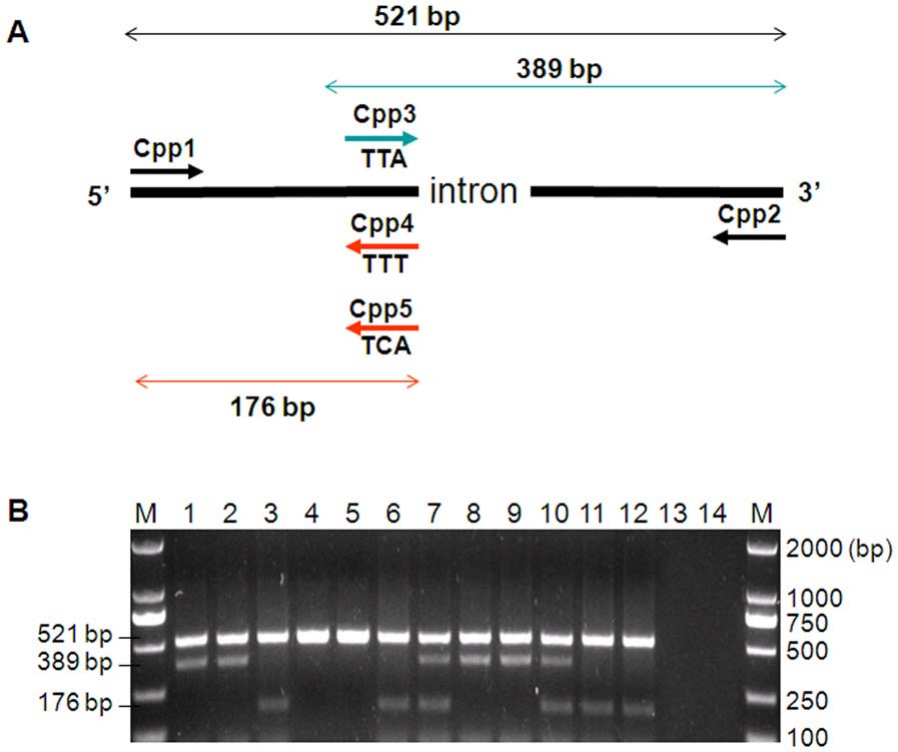
Allele-Specific PCR (AS-PCR) for *kdr* genotyping. **A**: Schematic representation of the primer location and predicted size of PCR products. Cpp1–Cpp5 indicate PCR primers in which sequences are reported in Table 5. Primer pair Cpp2 and Cpp3 amplifies a 389 bp fragment for the wildtype susceptible allele (for codon TTA). Primer pair Cpp1/Cpp4 yields a 176 bp fragment for resistant L1014F allele (codon TTT). Similarly, primer pair Cpp1/Cpp5 leads to amplification of a 176 bp fragment diagnostic to the L1014S resistant allele (for codon TCA). **B**: An example of AS-PCR gel. Two PCR reactions were run in parallel for each specimen (lanes 1 and 2 for specimen 1; lanes 3 and 4 for specimen 2…). Samples in lanes 1, 3, 5, 7, 9 and 11 were amplified with the primers Cpp1, Cpp2, Cpp3 and Cpp4 to detect wildtype L1014 (TTA) and resistant L1014F (TTT) alleles, whereas samples in lanes 2, 4, 6, 8, 10 and 12 were amplified with primers Cpp1, Cpp2, Cpp3 and Cpp5 to detect wildtype L1014 (TTA) and resistant L1014S (TCA) alleles. Lanes 13 and 14 were negative control. Genotype results: specimen 1: TTA/TTA; specimen 2: TTT/TTT; specimen 3: TCA/TCA; specimen 4: TTA/TTT; specimen 5: TTA/TCA; and specimen 6: TTT/TCA.

Based on the sequence data, we developed a *kdr* mutation diagnostic method using allele specific polymerase chain reaction (AS-PCR). In this method, a common 521-bp fragment was designed for both resistant and susceptible genotypes (Fig. 1). The discriminating fragments were a 389-bp fragment for wildtype (codon TTA) allele and a 176-bp fragment for mutant (codons TTT and TCA) alleles (Fig. 1). Therefore, genotype scoring was made on the basis of presence/absence of 389 bp and 176 bp fragments. The presence of single fragment indicates homozygous genotypes whereas the presence of both fragments stands for a heterozygote. Among the 97 sequenced specimens that were subjected to AS-PCR, 77 (79.4%) were successfully amplified by AS-PCR and 20 samples (20.6%) failed to amplify after 3 repeated PCR amplifications.

**Figure 2:**
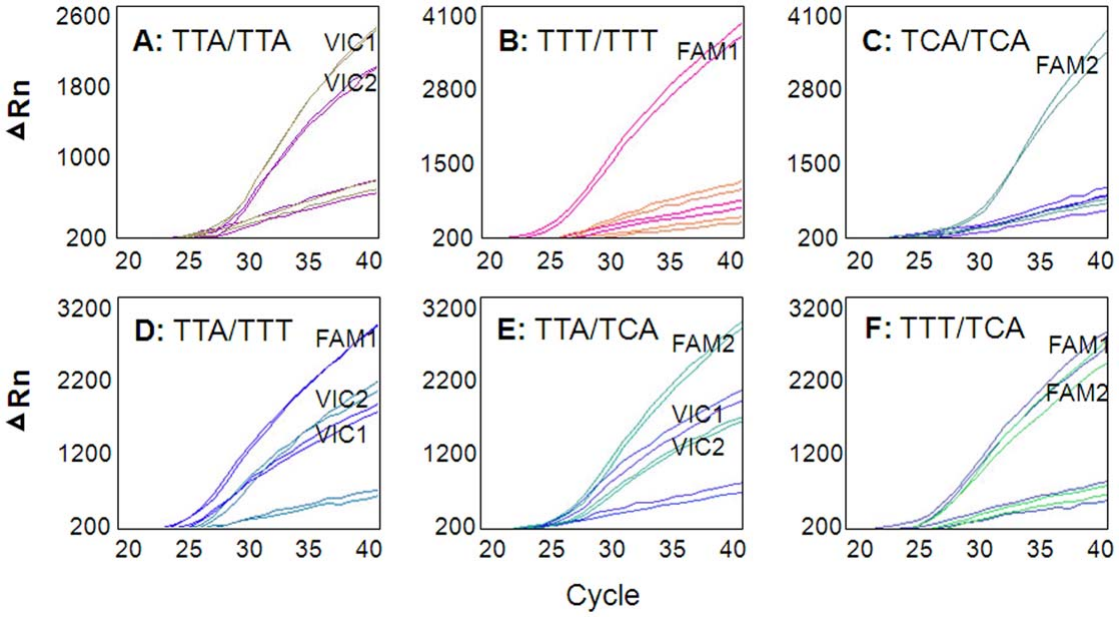
Real-time TaqMan method for genotyping *kdr* mutations at codon L1014 of *para*- sodium channel gene in *Culex pipiens pallens*. Genotype of specimens: A (TTA/TTA); B (TTT/TTT); C (TCA/TCA); D (TTA/TTT); E (TTA/TCA); and F (TTT/TCA).

Due to the high failure rate in PCR amplification of the AS-PCR method, we further tested a real-time TaqMan probe assay (refer to TaqMan method hereafter). Two TaqMan real-time reactions were performed in parallel for each specimen so that L1014F allele and L1014S allele can be diagnosed. In each reaction, a substantial increase in VIC fluorescence indicated a homozygous wildtype genotype, a substantial increase in FAM fluorescence indicated a homozygous mutant genotype and an increase in both signals indicated a heterozygote (Fig. 2).

Table 2 summarizes the genotyping results of direct DNA sequencing, AS-PCR and TaqMan method. Using the *kdr* gene sequence data as a gold standard, AS-PCR method showed 93.5% sensitivity and 95.0% specificity among those amplified samples (note that 20.6% samples were not amplified). The TaqMan method yielded 100% amplifications, 96.9% sensitivity and 99.4% specificity. The positive predictive value of the TaqMan method was 96.9%, substantially higher than the AS-PCR method (74.2%). Both the TaqMan and AS-PCR methods had similar negative predictive values (98.9% for AS-PCR and 99.4% for TaqMan method), the TaqMan method had a higher Kappa value (K = 0.963) than the AS-PCR method (K = 0.803), indicating a higher concordance between direct DNA sequence data and results from the TaqMan method. Therefore, for *kdr* allele frequency examination described in subsequent sections, we used the TaqMan method.

**Table 2:** Sensitivity and specificity of allele-specific PCR (AS-PCR) and real-time TaqMan methods in reference to allele sequence data for identification of *kdr* genotypes in *Culex pipiens pallens*.

	Detection methods		
	Sequencing (n = 97)	AS-PCR (n = 77)	TaqMan (n = 97)
Genotype frequency			
TTA/TTA	37.1	37.7	37.1
TTT/TTT	25.8	29.9	25.8
TTA/TTT	16.5	14.3	16.5
TCA/TCA	4.1	1.3	3.1
TTA/TCA	8.2	11.7	9.3
TTT/TCA	8.2	5.2	8.2
Sensitivity (%)	-	93.5	96.9
Specificity (%)	-	95.0	99.4
Positive Predictive Value (%)	-	74.2	96.9
Negative Predictive Value (%)	-	98.9	99.4
Kappa (95% CI)	-	0.803 (0.734–0.872)	0.963 (0.933–0.992)

The 97 *Cx. pipiens pallens* individuals used in this assay were from Wuxi population.

### Distribution of *kdr* allele frequencies in natural populations from eastern China

Using the TaqMan method and all individuals tested in susceptibility bioassay, the *kdr* allele frequencies in six *Cx. pipiens pallens* populations were examined. Table 3 summarized the frequency of *kdr* wildtype (L1014) and two mutations (L1014F and L1014S) in relation to survival phenotype in the deltamethrin susceptibility bioassay. We found that the wildtype L1014 allele frequency was significantly higher in those individuals killed by exposure to 0.05% deltamethrin in all six populations (Paired t-test, t = 11.40, d.f. = 5, P<0.0001). In contrast, the L1014F allele frequency was significantly higher in the surviving individuals than those dead individuals in all six populations (Paired t-test, t = 13.18, d.f. = 5, P<0.0001). However, the L1014S allele frequencies were similar between the dead and surviving individuals (Paired t-test, t = 1.65, d.f. = 5, P = 0.16). These data suggest that L1014F allele was associated in resistance to deltamethrin in *Cx. pipiens pallens* mosquitoes, but the role of L1014S allele in deltamethrin resistance is not clear.

**Table 3:** Frequencies (in percentage) of *kdr* alleles in relation to mosquito survival phenotype determined by the deltamethrin susceptibility bioassay in six *Culex pipiens pallens* populations from China.

Population	24 hr post exposure survival	Sample size (n)	Frequency of			Population frequency of resistant alleles (TTT+TCA)
			TTA (L1014)	TTT (L1014F)	TCA (L1014S)	
Tangkou	Alive	79	13.9	79.8	6.3	79.8
	Dead	20	45.0	40.0	15.0	
Qingdao	Alive	69	11.6	80.4	8.0	69.2
	Dead	35	68.6	20.0	11.4	
Huaibei	Alive	54	17.6	75.0	7.4	60.4
	Dead	42	67.9	20.2	11.9	
Weishan	Alive	39	26.9	60.3	12.8	50.5
	Dead	54	65.7	23.2	11.1	
Wuxi	Alive	33	13.6	74.3	12.1	50.5
	Dead	64	68.0	19.5	12.5	
Nanjing	Alive	22	18.2	81.2	0	40.3
	Dead	81	71.0	29.0	0	

**Figure 3:**
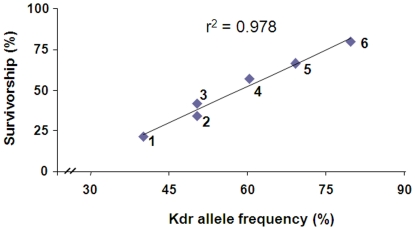
Relationship between mosquito survivorship in WHO insecticide susceptibility bioassay and frequency of *kdr* alleles (L1014F and L1014S) combined of *Culex pipiens pallens*. Numbers in the figure corresponds to the following sample localities: 1, Nanjing; 2, Wuxi; 3, Weishan; 4, Huaibei; 5, Qingdao; and 6, Tangkou.

The frequency of L1014F and L1014S alleles varied significantly among the six populations (P<0.0001) (Table 3), ranging from 40.3% (Nanjing population) to 79.8% (Tangkou population). When the survival rates were plotted against the frequencies of *kdr* alleles (L1014F alone, L1014S alone, or L1014F and L1014S combined), the levels of correlation also varied. We found the correlation between L1014S frequencies and survival rates to be poor, with a r^2^ value of 0.110. In comparison, the correlation between L1014F frequencies and survival rates was significantly better, with a r^2^ value of 0.870 (P<0.0001). Interestingly, the combined frequencies of L1014F and L1014S showed the best correlation with the survival rates, with a r^2^ value of 0.978 (P<0.0001) (Fig. 3). These data indicate the combined frequency of L1014F and L1014S is a better indicator of resistance in field mosquito populations than the frequency of either L1014F or L1014S alone.

### Frequency of L1014F and L1014S mutations in response to deltamethrin selection

**Figure 4:**
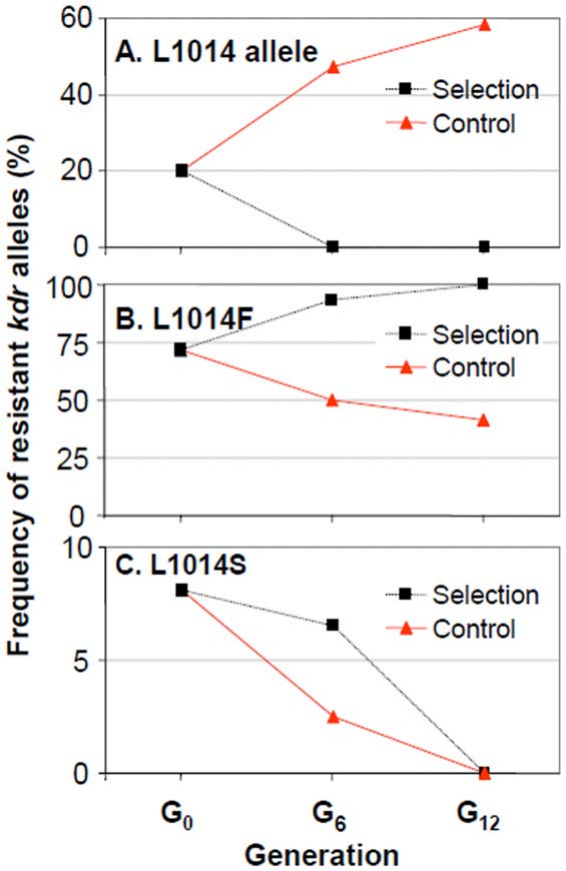
Dynamics of *kdr* allele frequency in response to deltamethrin selection. **A**: wildtype L1014 allele frequency was significantly decreased under deltamethrin selection, but significantly increased in the absence of selection; **B**: L1014F allele frequency was significantly increased under selection, but significantly decreased when no insecticide selection pressure was available; and **C**: L1014S allele frequency was decreased regardless of deltamethrin selection.

To further discern the role of L1014F and L1014S mutations in resistance to deltamethrin, we examined the response of these two mutations to deltamethrin selection in Tangkou *Cx. pipiens pallens* population. After six generations of selection, the 50% lethal concentration (LC_50_) was increased by 2.77 folds, and by 28.24 folds at generation 12 (Table 4). In the control population without insecticide selection, the LC_50_ value was constantly reduced at about 0.01 ppm. We observed interesting dynamics of the three *kdr* alleles in the population subjected to insecticide selection and the control population (Fig. 4). In the population with deltamethrin selection, the wildtype L1014 allele became extinct after six generations, whereas in the control population L1014 allele frequency was increased significantly from 20.2% to 47.5% (χ^2^ = 24.99, d.f. = 1, P<0.0001) after six generations, and to 58.3% after 12 generations of selection (P<0.0001) (Fig. 4A), demonstrating that the wildtype allele frequency increases in the absence of insecticide selection, likely due to a fitness advantage of susceptible genotype in the absence of insecticide selection pressure. On the other hand, frequency of resistant allele L1014F increased from 71.7% to 93.5% after six generations of deltamethrin selection (χ^2^ = 31.44, d.f. = 1, P<0.0001) and became fixed after 12 generations of selection (Fig. 4B). In the control population, the L1014F allele frequency declined monotonically (χ^2^ = 14.29, d.f. = 1, P<0.001). Interestingly, L1014S allele decreased from initial frequency of 8.1% and became completely absent after 12 generations in both the population subjected to selection (χ^2^ = 11.45, d.f. = 1, P<0.001) and the control population (χ^2^ = 8.59, d.f. = 1, P<0.01) (Fig. 4C). These results suggest that L1014F mutation is associated with deltamethrin resistance in *Cx. pipiens pallens* population, and but the L1014S mutation is not directly involved in deltamethrin resistance.

**Table 4:** Values of 50% lethal concentration (LC_50_) in Tangkou population of *Culex pipiens pallens* in response to deltamethrin selection.

Treatment	Generation	LC_50_ (ppm)	95% confidence interval	Slope	Resistance ratio*
	Parental (G_0_)	0.0204	0.0144–0.0289	1.2179	1.00
Selection	G_6_	0.0565	0.0413–0.0774	1.5672	2.77
	G_12_	0.5761	0.4858–0.6832	2.5899	28.24
Control	G_6_	0.0101	0.0084–0.0121	2.3149	0.49
	G_12_	0.0098	0.0081–0.0118	2.2160	0.48

*Resistance ratio is the ratio of LC_50_ of the test population to LC_50_ of the initial parental (G_0_) population.

## Discussion

Insecticide resistance represents the most important obstacle to insecticide-based vector control approach. Accurately monitoring resistance status is essential to guide the rational use of insecticides and resistance management. In the present study, we examined the nucleotide diversity of *kdr* gene in *Cx. pipiens pallens* mosquitoes in China, established molecular detection methods for the two key mutations associated with resistance, and examined the evolutionary dynamics of the two *kdr* alleles. We further determined that the combined frequency of L1014F and L1014S mutations was significantly associated with resistance measured as survival rate after exposure to a discriminating dose of deltamethrin.

Similar to *A. gambiae* in Africa [22], we identified two mutations (L1014F and L1014S) in the *kdr* gene in *Cx. pipiens pallens* of China. Due to potentially different role of each mutation in insecticide resistance, the molecular diagnostic methods are required to distinguish these two mutations. Allele-specific PCR (AS-PCR) methods were reported for *kdr* detection in *Culex* mosquitoes [8], [14], but these methods detected only one form of resistance allele (L1014F). We therefore modified the AS-PCR method so that two forms of *kdr* alleles can be detected. Although the AS-PCR method is rapid and low-cost in terms of capital expenditure and running costs, we found a high failure rate in amplification and low positive predictive value, in relation to the DNA sequencing gold standard. The lack of amplification reliability of AS-PCR method has also been reported in *kdr* typing of *A. gambiae* and *A. arabiensis* by other researchers [22], [29]. Therefore, the AS-PCR method is less valuable for molecular diagnosis of *kdr* resistance in *Cx. pipiens pallens*. On the other hand, the TaqMan method performs excellently as evidenced by high amplification rate, high positive and negative predictive values with regards to the *kdr* gene sequence results. Therefore, we recommend the TapMan method for molecular diagnosis of *kdr* resistance.

Based on the strong positive correlation between L1014F mutation frequency and mosquito survivorship in mosquito susceptibility bioassay and the evolutionary dynamics of L1014F mutation in response to deltamethrin selection, we suggest the L1014F mutation, not the L1014S mutation, is the key mutation associated with deltamethrin resistance. This notion is supported by following evidences. First, a significantly higher L1014F mutation frequency was consistently detected in the mosquitoes that survived the exposure to the standard WHO deltamethrin test paper in all six populations that we examined. However, this phenomenon was not observed for the L1014S mutation. Second and more importantly, the frequency of L1014F mutation increased monotonically in the laboratory mosquito population subjected to deltamethrin selection, whereas L1014S frequency decreased in both control and selection populations. Although enhanced metabolism of pyrethroid insecticides (e.g. cytochrome P450-mediated metabolism of pyrethroids) also contributes to resistance [30], [31], [32], the high correlation coefficient between *kdr* allele frequency and survival rate of mosquitoes in insecticide susceptibility bioassay found in the current study populations in Eastern China and in Northern China by other investigators [14] suggests that *kdr* mutation screening is an excellent molecular markers for pyrethroid resistance monitoring in Northern and Eastern China.

The dynamics of *kdr* alleles in our laboratory populations subjected to insecticide selection supports the hypothesis that L1014F resistance mutation has a fitness cost in the absence of insecticide selection pressure. In the original Tangkou population, the L1014F mutation frequency was 71.7%. Without deltamethrin selection, the L1014F mutation frequency was reduced to 41.7% after 12 generations. With insecticide selection pressure, its frequency increased rapidly and became fixed after 12 generations. While resistance alleles provide advantages in the presence of insecticides, insecticide-resistance genotype often exhibited lower fitness in comparison to the susceptible genotype in the absence of selection pressure from the insecticides. This phenomenon has been demonstrated in a variety of insects, including *Colorado potato beetle* [33], *Plutella xylostella* [34], *Spodoptera exigua* [35], *pink bollworm* [36], *Anopheles gambiae* and *An. stephensi* [37], *Culex quinquefasciatus* [38], *Culex pipiens* [39], [40]. It is interesting to note that the frequency of L1014S mutation decreased monotonically in both control population and the population subjected to insecticide selection, suggesting that the L1014S mutation does not directly confer resistance to deltamethrin insecticide. The decrease of L1014S allele frequency should not result from genetic drift because our populations were consistently maintained in large population size (2,000 adult each generation). The low L1014S allele frequency observed in our field-collected *Cx. pipiens pallens* populations may be a consequence of mosquito exposure to low concentration of DDT and its metabolites in water and soil from past public health vector and agricultural pest control [41], [42]. L1014S mutation is known to be associated with a high level of resistance to DDT, but a low level of resistance to pyrethroids [8].

Our study has important implications for *Cx. pipiens pallens* mosquito control. The six populations that we studied exhibited high survival rate in the WHO standard insecticide susceptibility bioassay (ranging from 21.4 to 79.8%). Consistently with the bioassay results, we detected high *kdr* allele frequency in these populations (ranging from 40.3% to 79.8%). Therefore, our results raise a serious concern on the efficacy of pyrethroid insecticides in Eastern China. Further research is required to examine the spatial distribution of *kdr* alleles and temporal dynamics in relation to insecticide use, determine the efficacy of the insecticides in the field, and research for alternative vector control strategies that minimize the use of insecticides or greatly reduce the spread of insecticide resistance.

## Materials and Methods

### Mosquito sampling

**Figure 5:**
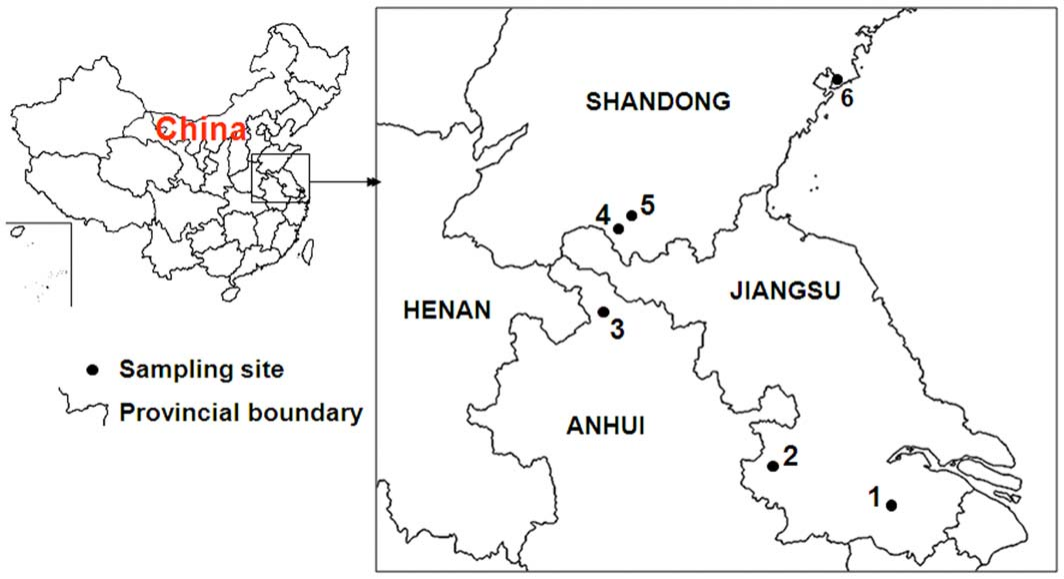
Map of China showing the distribution of mosquito sampling sites. 1, Wuxi (31°33′58.47″N, 120°18′9.88″E); 2, Nanjing (32°3′30.11″N, 118°47′47.28″E); 3, Huaibei (33°57′13.67″N, 116°47′56.95″E); 4, Weishan (34°48′25.40″N, 117°7′43.69″E; 5, Tangkou (34°52′34.97″N, 117°22′53.69″E); and 6, Qingdao (36°3′58.85″N, 120°22′57.98″E).

Six populations of *Cx. pipiens pallens* were used in this study, including Wuxi (Jiangsu province), Nanjing (Jiangsu province), Huaibei (Anhui province), Weishan (Shandong province), Tangkou (Shandong province) and Qingdao (Shandong province) (Fig. 5). Nanjing population was collected in July 2006 and has been reared in the insectary without exposure to any insecticides. The other five populations were collected from the natural habitats in July to August 2008. For each population, we collected several hundred larvae and pupa from more than 50 larval habitats per geographic site, using the standard 350 ml dippers. After identifying mosquito larvae to species by morphology, they were brought back to the insectary regulated at 28°C and 75% relative humidity and a constant light/dark (14 h∶10 h) cycle. The adults were used in deltamethrin resistance bioassay and *kdr* allele frequency determination.

### Insecticide susceptibility test

Non-bloodfed female *Cx. pipiens pallens* mosquitoes, 2 to 3 days post adult emergence, were tested for susceptibility to deltamethrin, using the standard WHO bottle bioassay with standard 0.05% deltamethrin-treated papers [43]. Adults directly reared from field-collected larvae were used for insecticide susceptibility test except that Nanjing population was a laboratory colony. For each population, about 100 female mosquitoes, 3 days post emergence, were tested. Specifically, the mosquitoes were equally divided into 4 groups (4 replicates). We used paraffin oil-treated papers without insecticide as control, which included 25 female mosquitoes for each population. After 1-hr exposure to diagnostic concentration of deltamethrin (0.05%), mosquitoes were transferred into insecticide-free tubes and maintained on 6% sucrose solution for 24 hrs. The number of dead and surviving mosquitoes was recorded, and they were preserved individually at −80°C for subsequent DNA analysis.

### Amplification and sequencing of sodium channel gene fragments in natural *Cx. pipiens pallens*

This experiment used *Cx. pipiens pallens* adult mosquitoes reared from larvae collected in Wuxi, Jiangsu province. Genomic DNA of individual mosquitoes was extracted using the method as previously described [44]. DNA samples were further confirmed to *Cx. pipiens pallens* using the previously described PCR method [45]. The region containing *kdr* mutations within the *para*-sodium channel gene was amplified by PCR, using the genomic DNA from individual mosquitoes as template. The PCR primers were designed based on the cDNA sequence of *Cx. quinquefasciatus para*-sodium channel gene alpha subunit (Genbank accession number BN001092). The amplicon includes the 1014 codon conferring resistance to pyrethroid in other mosquito species and an intron region. The PCR reaction contained 1 unit of Pyrobest Taq DNA polymerase (Takara, Japan), 10 ng genomic DNA, 0.4µM of primers Cpp1 and Cpp2 (see Table 5 for primer sequence), 0.2mM dNTPs, and the buffer provided by the polymerase supplier in a final volume of 50ul. Amplification was performed with the following cycling conditions: 30 cycles of 94°C for 40 s, 60°C for 50 s and elongation at 72°C for 40 s, followed by extension at 72°C for 8 min. PCR products were purified using the QIAquick PCR purification kit (Qiagen) and directly sequenced from both ends using ABI Big-Dye Terminator Cycle Sequencing Kit. PCR products from a total of 97 individuals were sequenced. The unique DNA haplotype sequences were deposited into GenBank (accession number GU198929 - GU198944, GU325775 - GU325777, GU339219 - GU339221). Sequences were aligned and analyzed using the ClustalW software [46] to determine polymorphism and synonymous or non-synonymous mutations.

**Table 5:** PCR primer and TaqMan probe sequences used in the present study.

Name	Sequence (5′ -> 3′)
PCR primer	
Cpp1	CCT GCC ACG GTG GAA CTT C
Cpp2	GGA CAA AAG CAA GGC TAA GAA
Cpp3	CCA CCG TAG TGA TAG GAA ATT TA
Cpp4	ACG CTG GAA TAC TCA CGA CA
Cpp5	ACG CTG GAA TAC TCA CGA CTG
*kdr*F	GTG TCC TGC ATT CCG TTC TT
*kdr*R	TTC GTT CCC ACC TTT TCT TG
TaqMan probe	
Wildtype	CTC ACG ACT AAA TTT C
FAM1	CAC GAC AAA ATT TC
FAM2	CAC GAC TGA ATT TC

### Development and validation of allele-specific PCR (AS-PCR) method for detecting *kdr* mutations

Because our study populations contained three forms of *kdr* alleles (wildtype, L1014F and L1014S) and previous AS-PCR method detects only two alleles (wildtype and L1014F) [14], we modified the AS-PCR method to differentiate the two mutations from the wildtype allele (Fig. 1A). Primer pair Cpp2 and Cpp3 (see Table 5 for primer sequences) amplifies a 389 bp fragment characteristic of wildtype susceptible allele (termed as L1014 allele for codon TTA), and primer pair Cpp1 and Cpp4 yields a 176 bp fragment representing a resistant allele (L1014F allele for codon TTT). Similarly, primer pair Cpp1 and Cpp5 amplifies a 176 bp fragment diagnostic to the L1014S resistant allele (for codon TCA). Therefore, the AS-PCR method includes two independent PCR reactions. The first reaction uses four primers (Cpp1, Cpp2, Cpp3 and Cpp4) to detect L1014 and L1014F alleles, and the second uses four primers (Cpp1, Cpp2, Cpp3 and Cpp5) to detect L1014 and 1014S alleles. The PCR condition was 1×PCR buffer, 0.25µM of Cpp1 and Cpp2, 0.5 µM of Cpp3 and Cpp4 (or Cpp5), 2mM of Mgcl_2_, 0.2 mM dNTP mix, and 2.5 U Taq Polymerase (Promega) in a final reaction volume of 50µl. The PCR cycling condition was 5 min at 94°C, followed by 40 cycles of 1 min at 94°C, 2 min at 50°C and 2 min for 72°C and 10 min extension at 72°C. The DNA fragments were analyzed by electrophoresis on 1.5% agarose gel and visualized by ethidium bromide staining. DNA from all 97 mosquitoes used in the above *kdr* gene sequencing from natural habitats in Wuxi was tested for amplification success rate, sensitivity and specificity of the AS-PCR method.

### Detection of *kdr* mutations using TaqMan probe

**Figure 6:**
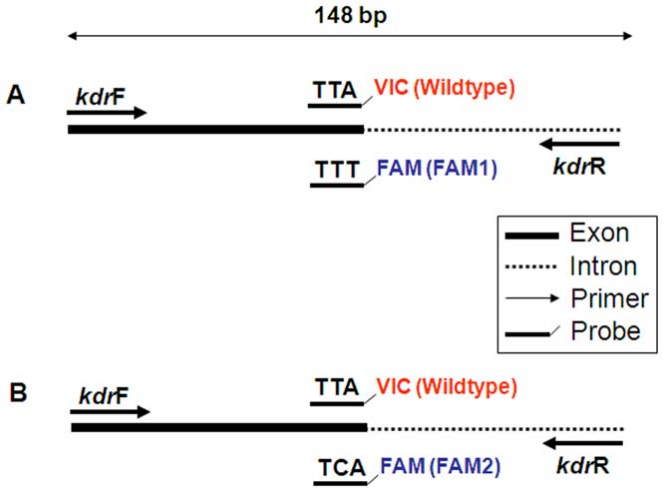
Schematic diagram showing the design of primers and probes of TaqMan method for molecular diagnosis of *kdr* mutations. Solid and dashed lines represent exon and intron region of the *para*-sodium channel gene, respectively. Two TaqMan real-time PCR reactions are required in parallel for one specimen to differentiate TTT and TCA mutations in codon 1014. **A**: Primers (*kdr*F and *kdr*R) and probes (VIC and FAM1) are for detection of wildtype (TTA) and *kdr* mutation (TTT) at codon L1014. **B**: Primers (*kdr*F and *kdr*R) and probes (VIC and FAM2) are for detection of wildtype (TTA) and *kdr* mutation (TCA) at codon L1014.

TaqMan method of *kdr* mutation was developed following a previously described method [47]. Briefly, we designed forward and reverse primers and three minor groove binding probes (Applied Biosystems), using the Primer Express™ Software (version 2.0). The primer and TaqMan probe sequences are listed in Table 5. Probe “Wildtype” was labeled with VIC™ to detect wildtype L1014 allele (codon TTA at site 1014) (Fig. 6). Probes “FAM1” and “FAM2”, labeled with 6-FAM™, are for detection of the L1014F (codon TTT) and L1014S (codon TCA) alleles, respectively. In order to differentiate the two resistant alleles (L1014F and L1014S), two TaqMan real-time reactions were performed in parallel. The first reaction uses primers *kdr*F and *kdr*R, and probes Wildtype and FAM1 to detect L1014F allele; the second reaction uses primers *kdr*F and *kdr*R, and probes Wildtype and FAM2 to detect L1014S allele (Fig. 6). Each reaction included at least one negative control (double deionized molecular grade water). All samples and controls were amplified in triplicate.

Each PCR reaction used 10 ul of mixture consisting of 5 ng of mosquito genomic DNA, 5.0 µl of 2×PCR Mastermix (Thermo Scientific AB-0575-DC, Abgene, UK), 900 nM of each primer and 200 nM of each probe. The cycling conditions were 50°C for 2 min for carry-over inactivation, and 95°C for 10 min, followed by 40 cycles of 92°C for 15 sec and 60°C for 1 min. Samples were run on the ABI PRISM 7900 HT sequence detection system in 384-well format (Applied Biosystems). The system automatically monitors the increase in VIC and FAM fluorescence intensities in real time at each cycle. The threshold cycle (*Ct*) value of each PCR reaction was calculated using the software provided by the manufacturer. The same 97 mosquitoes used in the above *kdr* gene sequencing and AS-PCR method were tested using the Taqman method to determine its sensitivity and specificity.

### Deltamethrin selections

To determine the dynamics of *kdr* alleles in response to insecticide selection, *Cx. pipiens pallens* population collected in Tangkou, Shandong Province, was subjected to deltamethrin selection at larval stage for a total of 12 generations in the laboratory. The concentration of deltamethrin (Jiangsu Yangnong Chemical Group Co.) used in the selection caused about 50% mortality among the fourth-instar larvae. For each population and at each generation, we maintained 3 larval trays, about 1000 larvae per tray, for the selected line and control line. The selected line normally incurred 50% larval mortality. Therefore, the adult population size for the selected line is at about 1000 individuals, and at least 2,000 individuals for the control line. Genetic drift is negligible under such a population size for limited number of generations (12). At generations 6 and 12, 50% lethal concentrations (LC_50_) were determined by bioasay. Briefly, about 250 fourth-instar larvae, divided into 5 groups, were exposed to 5 different concentrations of deltamethrin for 24 hr. Mortality of the control population was measured using 50 fourth-instar larvae water without any insecticides. The number of dead and surviving mosquitoes was recorded after 24 hr. LC_50_ was calculated using the Probit analysis [48] and Abbott's correction for mortality rate in the control population [49]. The *kdr* allele frequencies were examined in 100 randomly selected adult mosquitoes at generations 6 and 12 of Tangkou population under deltamethrin selection, using the Taqman method. Similarly, *Kdr* allele frequencies were examined in 100 mosquitoes at generations 6 and 12 in the same Tangkou population maintained in the laboratory without any insecticide selection.

### Statistical analysis

To determine among-population difference in mosquito mortality rate in WHO insecticide susceptibility bioassay, univariate analysis of variance was conducted using arcsin transformation. *Kdr* allele frequency in six natural mosquito populations was calculated, and statistical difference among populations was examined using the *t* test. Linear regression analysis was conducted to determine the correlation coefficient between *kdr* allele frequency and bioassay-based mosquito survivorship in *Cx. pipiens pallens*.
